# Transportability of confined field trial data from cultivation to import countries for environmental risk assessment of genetically modified crops

**DOI:** 10.1007/s11248-015-9892-6

**Published:** 2015-07-03

**Authors:** Shuichi Nakai, Kana Hoshikawa, Ayako Shimono, Ryo Ohsawa

**Affiliations:** Monsanto Japan Limited, Kyobashi Soseikan Building 6F, 2-5-18, Kyobashi, Chuo-ku, Tokyo, 104-0031 Japan; Faculty of Science, Toho University, 2-2-1 Miyata, Funabashi, Chiba 274-8510 Japan; Faculty of Life and Environmental Sciences, University of Tsukuba, 1-1-1 Tennodai, Tsukuba, Ibaraki 305-8572 Japan

**Keywords:** Data transportability, Environmental risk assessment, Genetically modified crops, Confined field trial, Weediness potential

## Abstract

Requirement of in-country confined field trials for genetically modified (GM) crops prior to unrestricted release is well-established among countries with domestic regulations for the cultivation approval of GM crops. However, the requirement of in-country confined field trials is not common in countries where the scope of the application does not include cultivation. Nonetheless, Japan and China request in-country confined field trials for GM crops which are intended only for use as food, feed and processing. This paper considers the transportability of confined field trial data from cultivation countries (e.g. United States, Canada, and South American countries) to import countries like Japan for the environmental risk assessment of GM crops by reviewing: (1) the purpose of confined field trial assessment, (2) weediness potential, defined as “an ability to establish and persist in an unmanaged area that is frequently disturbed by human activity”, of host crops, and (3) reliability of the confined field trial data obtained from cultivation countries. To review the reliability of the confined field data obtained in the US, this paper describes actual examples of three confined field trials of approved GM corn events conducted both in the US and Japan. Based on the above considerations, this paper concludes that confined field data of GM corn and cotton is transportable from cultivation countries to importing countries (e.g. from the US to Japan), regardless of the characteristics of the inserted gene(s). In addition, this paper advocates harmonization of protocols for confined field trials to facilitate more efficient data transportability across different geographies.

**Electronic supplementary material:**

The online version of this article (doi:10.1007/s11248-015-9892-6) contains supplementary material, which is available to authorized users.

## Introduction

Between 1996 and 2013, global production of genetically modified (GM) crops increased from 1.7 million hectares to over 175 million hectares, and the number of countries in which GM crops are produced increased from six to 27 (James 2013). The vast majority of the GM crops consist of soybean, corn, cotton, and canola, although modified forms of other crops, such as alfalfa, sugar beet, and papaya have also been developed and commercialized (USDA Global Agricultural Information Network (GAIN) 2013).

Although GM food crops are not commercially cultivated in Japan, Japan is one of the world’s largest importers of agricultural products intended for food and feed that have been produced using GM crops (USDA Global Agricultural Information Network (GAIN) 2013). Japan imports approximately 15 million metric tons of corn and three million metric tons of soybeans from around the world each year, approximately three-quarters of which are produced using GM crops. Although Japan’s self-sufficiency of rice is 96 %, its self-sufficiency for grains overall is only 28 % (MAFF 2015). Due to its high dependence on grain supplied from foreign countries (>70 %) and high penetration of GM crops in major crops such as soybean, corn and cotton, GM crops have already become essential elements to securing Japan’s food supply.

Japan ratified the Cartagena Protocol on Biosafety in 2003. To implement the Protocol, Japan adopted the “Law Concerning the Conservation and Sustainable Use of Biological Diversity through Regulations on the Use of Living Modified Organisms” also called the “Cartagena Law” in 2004. Under the “Cartagena Law”, the Ministry of Agriculture, Forestry and Fisheries (MAFF) and the Ministry of Environment (MOE) grant joint approvals for cultivation or for the use of GM crops as food and feed. A joint MAFF and MOE expert panel carries out an environmental risk assessment (ERA) to determine the potential for adverse effects on biodiversity, focusing on “the influence of competition on native wild species by living modified organisms (LMO) (competitive superiority)”, “the influence of LMO which produces harmful substances (potential production of harmful substance)”, and “the influence of LMO hybridizing with native wild species (crossability)” (Japan Biosafety Clearing House (J-BCH) 2014a).

As with other regulatory systems around the world, Japan’s biotechnology review system could benefit from leveraging their cumulative data and experiences. One of the areas necessitating further consideration is Japan’s in-country confined field trial requirement prior to approval for the purposes of food, feed, or processing (FFP). Despite the fact that GM crops are not intended to be commercially grown in Japan, GM crop developers are required to perform in-country confined field trials to address potential environmental impacts from the unintended growth of GM crops as a result of unlikely events such as spillage during transportation and contamination of conventional planting seeds with GM seeds. Currently only Japan and China require in-country field trials for GM crops intended only for import use as FFP (USDA Global Agricultural Information Network (GAIN) 2013).

In Dec. 2014, MAFF announced that it would begin accepting data from confined field trials carried out in cultivation countries for ERA of GM corn with familiar traits (Director-General of Food Safety and Consumer Affairs Bureau et al. 2014). To be recognized as familiar traits, however, the mode of action (MOA) needs to be thoroughly understood as evidenced by a peer reviewed publication or a national investigative commission. Also, the efficacy of the trait being assessed needs to be comparable to that of the other traits which have already been approved. The major reason why MAFF does not accept transportable data for ERA of GM corn with novel traits is that the GM corn may exhibit different growth under different environmental conditions such as soil type and weather conditions in Japan. Currently MAFF does not accept transportable data for GM cotton, canola and soybean due to limited information on the growth of cotton in the natural environment in Japan, the relatively high weediness potential of canola, and presence of a cross compatible or sexually compatible wild relative of soybean in Japan. Weediness potential mentioned here is usually defined as “an ability to establish and persist in an unmanaged area frequently disturbed by human activity”.

This paper considers the transportability of confined field trial data obtained in cultivation countries (e.g. the US, Canada, and South American countries) to import countries such as Japan for ERA of GM crops regardless of the characteristics of inserted gene(s) by reviewing: (1) the purpose of confined field trial assessment, (2) weediness potential of host crops, and (3) reliability of the confined field trial data obtained in cultivation countries. Based on the above considerations, this paper concludes that confined field data of GM corn and cotton is transportable from cultivation countries to importing countries (e.g. from the US to Japan), regardless of the characteristics of the inserted gene(s).

## Purpose of confined field trial assessment

To consider the potential transportability of confined field trial data of GM crops from cultivation to import countries, it is important to have a clear understanding of the purpose of a confined field trial. While GM crops may exhibit different growth under different environmental conditions due to soil type or weather conditions, the purpose of confined field trials for ERA is not to describe GM crops in as much detail as possible in each of different environmental conditions. Rather, the purpose of confined field trials for GM crops is to identify whether any unintended and adverse changes occurred related to ERA assessment endpoints (Raybould 2007).

Assessment endpoints are defined during the problem formulation process, which comprises the initial step of an ERA (US Environmental Protection Agency 1998). Although assessment endpoints vary depending on the outcome of the problem formulation process, widely accepted, globally recognized assessment endpoints for the ERA of a GM crop may include: the reduction of abundance of a valued species through either competition with GM crops or any wild relatives which may receive the transgene via gene flow, or harmful impact of the introduced gene (Chandler and Dunwell 2008; Lu 2008). Similarly, in Japan GM crops are assessed for competitive superiority and potential production of harmful substances in order to determine whether the GM crop demonstrates the properties of invasive weeds, thereby causing negative impacts to the population size of wild plants, or other adverse ecological impacts [i.e. adverse effect on non-target organisms (NTO)] (Japan Biosafety Clearing House (J-BCH) 2014a).

A theoretical scenario by which harm may arise from the introduction of a GM crop that reproduces by seed has been suggested by Raybould (2010): (1) The GM crops produce seeds. (2) Seeds disperse to non-agricultural habitats. (3) The crop establishes in the non-agricultural habitats. (4) The crop forms a self sustaining population. (5) The population increases in abundance. (6) Increased abundance of the crop reduces the abundance of a valued species (ecological harm). In general, in confined field trials for GM crops, none of these steps are tested directly; instead, the GM crop is compared with non-GM control with genetically similar backgrounds to identify any unintended changes related to weediness potential (Raybould et al. 2012). When statistically significant differences in morphological phenotypes are detected in confined field trials, the primary question is whether the differences imply any significance for the assessment endpoints (Roberts et al. 2014). While environmental factors may affect the phenotype, there is no scientific evidence suggesting that environmental factors would reveal a difference between the GM crop and non-GM control (Garcia-Alonso et al. 2014).

Both the US and Japan rely on the concept of familiarity (Horak et al. 2007, 2015) when interpreting statistical differences identified between the GM crop and a conventional control in confined field trials. As described by the Organisation for Economic Co-operation and Development (OECD), familiarity is a concept coming from the knowledge and experience gained over time (Nickson and Horak 2006; OECD 1993). Familiarity considers the nature of the crop that was modified, the characteristics of the trait that was introduced, the likely receiving environment for the GM crop, and the likely interactions between these (OECD 1993; Nickson and McKee 2002). If statistical differences are detected between the GM crop and its conventional control, the mean values for the GM crop are then assessed in relation to the range of values of the reference varieties or range of literature values in the context of known values common for the crop. If the mean value for the GM crop is outside the range of values common for the crop, or if these ranges are not available, the detected differences are then assessed to determine whether they could alter weediness potential.

Weed scientists have already developed lists of characteristics that are observed in many common weeds, including seed dormancy, ability to compete interspecifically, adaptation for short and long distance seed dispersal, high seed output in favorable environments, and seed output throughout the growing region (Lingenfelter and Hartwig 2003; Anderson 1996). Only in cases where there is a lack of familiarity with the unmodified crop in the receiving environment, or where evidence suggests the GM crop is substantially different from the unmodified crop with respect to survival and persistence as assessed in the confined field trial, would the collection of additional experimental data above and beyond that typically obtained from confined field trial endpoints be necessary to inform the assessment (Roberts et al. 2014).

As noted above, the purpose of confined field trials for ERA is not to describe GM crops in as much detail as possible, but to identify whether any unintended and adverse changes occurred related to ERA assessment endpoints (Raybould 2007). Additionally, for host crops which exhibit low weediness potential and have no sexually compatible wild relatives a primal purpose of confined field trial is to identify any potential increases in weediness potential.

## Weediness potential of host crops

Understanding the weediness potential of host crops is also important to consider the transportability of confined field trial data of GM crops. Modern corn cannot survive as a weed due to intensive selection during the domestication of corn. Through the domestication of corn, traits often associated with weediness potential, such as seed dormancy and a dispersal mechanism have been lost which limit its ability to form reproducing populations outside of cultivation. For example, the corn ear is enclosed with husks; consequently, seed dispersal of individual kernels is limited. Even if individual kernels of corn were distributed within a field or along transportation routes from the fields to storage or processing facilities, sustainable volunteer corn populations are typically not found growing in fence rows, ditches, and road sides. As established in the literature, corn is poorly suited to survive without human assistance and is not capable of surviving as a weed (Baker 1965; Keeler 1989; Galinat 1988).

In Japan, MAFF investigated corn growth around five ports, six landing silos, and 10 feed mills across the country from May to September 2013; only one corn plant was found in transportation routes from unloading silos to a feed mill (MAFF 2014).

Soybeans do not occur as sustainable populations outside of cultivation in North America (OECD 2000). *Glycine soja* is a sexually compatible wild species grown in several countries in Asia (OECD 2000; Numata and Yoshizawa 1975; The Weed Science Society of Japan 1991). However, pollen-mediated gene flow between cultivated soybean (*G. max*) and *G. soja* is limited because they are both considered typical autogamous (self-pollinating). In addition, the rate of cross-pollination within these species has been reported as 0.30–3.62 % for soybean (Beard and Knowles 1971) and 2.3 % on average for *G. soja* (Kiang et al. 1992).

MAFF also investigated the growth of soybean outside cultivation areas for several years. From 2009 to 2012, 10 ports and the surrounding five km areas were investigated for soybean volunteer presence. As a result of MAFF’s investigation, an annual maximum of 16 soybean plants (two GM soybean plants) were discovered in 2009 (MAFF 2011a, b, 2012, 2013).This result clearly indicates low survivability of imported soybean grains spilled from trucks during transportation.

Cotton is another commodity crop that has lost the majority of traits that may contribute to weediness potential from its wild progenitor through domestication. Cotton is cultivated in Japan primarily as a decorative plant and not for commercial purposes, and there have been no reports of cotton becoming self-sustaining outside of cultivation in Japan.

Canola (*Brassica napus*) grows along roadsides, industrial sites and other places that are disturbed on a regular basis (OECD 1997). In Japan, there are reports indicating that canola is cultivated in flood plains along rivers (Shimizu et al. 2001; Ministry of Land Infrastructure Transport and Tourism 2015) and can grow around off-loading harbors and transportation routes (Japan Biosafety Clearing House (J-BCH) 2013).

Canola is generally regarded as an opportunistic species that is adapted to take advantage of temporary conditions such as disturbed areas (CFIA 2005). It is generally known that canola volunteer populations will not persist when grown in an undisturbed natural environment due to competition with perennial grasses, tree species and perennial shrubs in forests (OECD 1997). Unlike the introduced dandelion species (*Taraxacum* spp.) and tall goldenrod (*Solidago altissima*) (Hattori 2002; Ogawa 2002) in Japan, canola is not listed as an invasive alien species that can specifically affect the ecosystem. In Europe, canola is also not generally regarded as an environmentally hazardous colonizing species (EC 2000). Moreover, reports indicate that canola is not invasive of undisturbed natural habitats (Crawley et al. 1993; European Commission 2000; Hall et al. 2005). In addition, it has been reported that populations of canola established on undisturbed ground tend to go extinct after only a few years (Crawley and Brown 1995; Hall et al. 2005), suggesting it has low potential for causing ecological effects.

Therefore, although canola could grow as a volunteer in a frequently disturbed environment, the competitiveness under natural conditions (undisturbed environment) is very low and the possibility of forming invasive populations is considered to be low as well.

*B. rapa*, *B. nigra*, *Raphanus raphanistrum, Sinapis arvensis*, *B. juncea* and *Hirschfeldiaincana* are known as potentially sexually compatible relatives of canola, which exist in Japan. However, none is recognized as a wild species that should be protected under the Cartagena Law in Japan, primarily because *B. rapa* is a cultivar and other species were introduced to Japan (Shimizu et al. 2001; Nakai 2003; Tsunoda 2001).

Most of the commodity crops, including the four-mentioned above, have lost many of the weed-related traits of their wild progenitors through domestication (OECD Environment Directorate 2013). For these highly domesticated commodity crops except canola, strict similarity of environmental conditions is not necessary to consider transportability of confined field trial data to detect any changes related to invasive weediness potential. These weedy characteristics are often complex and encoded by many genes, and hence, these fundamental weediness characteristics are not considered to be expressed differently under different environmental conditions such as different soil type and weather conditions.

## Reliability of the confined field trial data obtained in cultivation countries

To further develop this proposal on transportability of confined field trial data, this paper reviewed the results of the confined field trials for three GM corn events conducted both in the US and Japan as case studies. The goal of reviewing these data is to illustrate how results from the US confined fields trials are relevant for conducting the ERA of GM crops for an import country like Japan.

Specifically, this paper reviewed ERA data for Lysine maize LY038, lepidopteran insect-protected corn MON 89034, and drought-tolerant corn MON 87460, all of which have previously been deregulated in the US and approved in Japan under the Cartagena Law. The submission documents for the three GM corn varieties are available in the websites of both United States Department of Agriculture (USDA) Animal and Plant Health Inspection Service (APHIS) and Japan Biosafety Clearing House (J-BCH) (APHIS 2015; J-BCH 2015).

Lysine maize LY038 was developed through the use of recombinant DNA techniques, to integrate the *cordapA* coding sequence into the maize genome. The *cordapA* sequence is under the control of the maize Glb1 promoter to direct expression of the *Corynebacterium glutamicum*-derived lysine-insensitive dihydrodipicolinate synthase (cDHDPS) enzyme predominantly in the germ, resulting in increased levels of lysine in grain for animal feed applications.

Lepidopteran insect-protected corn MON 89034 expresses Cry1A.105 and Cry2Ab2 insecticidal proteins and is protected from feeding damage caused by European corn borer (*Ostrinia nubilialis*) and other lepidopteran insect pests. Cry1A.105 is a modified *Bacillus thuringiensis* (*Bt*) Cry1A protein with 93.6 % overall amino acid sequence homology to the Cry1Ac protein. Cry2Ab2 is also a *Bt* (subsp. *kurstaki*) protein. The combination of the Cry1A.105 and Cry2Ab2 insecticidal proteins in a single plant provides broad spectrum of insect control and offers an enhanced insect-resistance management tool.

Drought-tolerant corn MON 87460 expresses a cold shock protein B (CSPB) produced from the inserted *B. subtilis*-derived gene. In bacteria, the CSPB protein helps preserve normal cellular functions during certain stresses by binding cellular RNA and unfolding non-translatable secondary structures affecting RNA stability and translation. During product development, MON 87460 exhibited reduced yield loss under water-limited conditions compared to conventional corn. Like conventional corn, MON 87460 is still subject to yield loss under water-limited conditions, particularly during flowering and grainfill periods when corn yield potential is most sensitive to stress as a result of disrupted kernel development (Monsanto Company 2009).

As summarized in the Table 1, confined field data were obtained from multiple locations and multiple years in the US. For example, phenotypic and agronomic data for Lysine maize LY038 were obtained at 10 and seven sites in 2002 and 2003, respectively, in Missouri, Illinois, Indiana, Iowa, and Nebraska (Monsanto Company 2004). These diverse locations provided a range of environmental and agronomic conditions representing major US corn-growing regions where commercial production of the GM crops would be expected. Notably, drought-tolerant corn MON 87460 was tested in more diverse field conditions such as (1) well-watered, (2) both well-watered and water-limited treatments established in the same field, or (3) water managed according to typical agronomic practices, which included typical amounts of supplemental irrigation at relevant sites. Because MON 87460 reduces yield loss under water-limited conditions, field studies were designed to evaluate the environmental consequences of MON 87460 performance across a broad range of soil moisture and environmental conditions (Sammons et al. 2014).

**Table 1:** Summary of the US field studies

Events	Number of field sites			Conventional varieties used to determine reference range
LY038	17 sites	10 sites (2002, US)		4 varieties
		7 sites (2003, US)		4 varieties
MON 89034	18 sites	9 sites (2004, US)		23 varieties in 2004
		9 sites (2005, US)	4 sites (study-1)	12 varieties in study-1 of 2005
			5 sites (study-2)	14 varieties in study-2 of 2005
MON 87460	31 sites	8 sites (2006, US)	Well-watered	19 varieties
		9 sites (2007, US)	Well-watered	11 varieties
		4 sites (2006/2007, Chile)	Well-watered and water-limited^a^	12 varieties
		5 sites (2007, US)	Well-watered and water-limited at 2 sites (study-1)	7 varieties
			Well-watered and water-limited at 3 site (study-2)^b^	12 varieties for well-watered and 4 additional varieties for water-limited
		5 sites (2006, US)	Typical agronomic conditions	15 varieties

^a^Four sites were evaluated with well-watered and water-limited treatments in Chile (Calera de Tango, Colina, Lumbreras and Quillota). The field site in Quillota did not meet the appropriate water stress treatments; thus, data for this site were not included in the statistical analysis

^b^Three sites were evaluated with well-watered and water-limited treatments in the US (Kansas, Nebraska and Texas). The field site in Texas was the only site to meet the inclusion criteria for both well-watered and water-limited treatments. Due to rainfall during the imposed water-limitation treatments at two sites in Kansas and Nebraska, the well-watered treatments met the inclusion criteria but the water-limited treatments did not. Thus, the water-limited treatment data from Kansas and Nebraska were not included in the statistical analysis

In Japan, data from a confined field trial obtained at a single location and a single year is accepted for both cultivation and import approval (Table 2). Furthermore, the tassels of GM corn are usually cut off or covered by paper bags because it is difficult to ensure sufficient isolation distance to limit cross-pollination with conventional corn varieties which grow in neighborhoods in Japan; while isolation distances can be established and managed in the US field trials. This measure to avoid cross-pollination in Japan makes it difficult to obtain reliable data from field trials for the ERA of GM crops.

**Table 2:** Summary of Japan field studies

Events	Number of field sites	Conventional varieties used to determine reference range
LY038	1 site	Minimum and maximum mean values of the non-GM controls used in the previous field trials of the following GM corn varieties: DLL25 (1998), NK 603 (2000), MON 863 (2000), MON 810 (1996, 2001), MON 88001 (2002), MON 88012 (2002), MON 88017 (2002), LY038 (2004), MON 89034 (2006), MON 87460 (2010), MON 87427 (2010)
MON 89034	1 site	
MON 87460	1 site (well-watered and water-limited)	

Regarding the data requirements for the ERA of GM corn and cotton, some differences exist between the US and Japan (Table 3). For example, “tolerance to low or high temperature of immature plants” and “the overwintering or over summering ability of the mature plant” are not requested for any GM crops in the US. However, it is usually the case that GM crops tested in the US are exposed to a wide range of field temperatures by testing the crop at multiple locations covering the major US corn-growing regions as described above, thereby effectively addressing these Japanese requirements. Additionally, ERAs that are science-based should be hypothesis driven, and therefore abiotic stress tolerance studies, including cold stress, are conducted in the US based on the characteristics of the inserted gene(s). For example, drought, cold, heat, and salt stress studies were conducted under controlled environmental conditions, such as greenhouses and growth chambers, for drought-tolerant corn MON 87460 in the US, because cold shock proteins are known to mitigate multiple abiotic stressors in both bacteria and plants (Castiglioni et al. 2008). Results support the conclusion that the abiotic stress tolerance of MON 87460 during young plant growth stages is not meaningfully different compared to conventional corn (Monsanto Company 2009). Consistent with a hypothesis driven approach for the ERA of GM crops, these comprehensive studies to confirm abiotic stress tolerance were not conducted for non-stress-tolerant events such as LY038 and MON 89034.

**Table 3:** Comparison of data requirement between the US and Japan for corn and cotton

Evaluation items	USDA^a^	Japan
Competitiveness		
Agronomic/phenotypic evaluation	✓	✓
Examples of data collected for corn^b^	Seedling vigor, Early stand count, Days to 50 % pollen shed, Days to 50 % silking, Stay green, **Ear height**, **Plant height**, Dropped ears, Stalk lodged plants, Root lodged plants, Final stand count, Grain moisture, **Test weight**	Uniformity of germination, Germination rate, Date of 50 % tasseling, Date of 50 % silking, Date of first flowering, Date of 50 % flowering, **Main stem height**, **Ear height**, Number of tillers, flag Leaf angle, Date of maturation, **Plant weight at harvest**, Grain shape, Grain color
Tolerance to low or high temperature of immature plants		✓
The overwintering or over summering ability of the mature plant		✓
Pollen morphology and viability	✓	✓
Examples of data collected for corn	**Pollen morphology**, **Pollen viability**, **Pollen diameter**	**Pollen morphology**, **Pollen viability**, **Pollen diameter** (by eye observation)
The production amount, seed shattering, dormancy and germination of harvested seed	✓	✓
Examples of data collected for corn	Yield, **Seed germination** **and dormancy assessments** at multiple temperature regimes	Number of grain-set ears, Ear length, Ear diameter, Number of grain rows, Number of grains per ear, 100 grain weight, Presence of shattering, **Germination rate of harvested seeds** at single temperature regime
Potential production of harmful substance		
Residual effects of substances which exist in the plant body and which will affect other plants after the death of the plant body		✓
Residual effects of substances which are secreted from roots and which affect other plants		✓
Substances which are secreted from roots and which affect microorganisms in soil		✓
Ecological interaction (observation)	✓	
Crossability	N/A	N/A

^a^United States Department of Agriculture

^b^Evaluation items in bold are common items between the US and Japan

Evaluation of “potential production of harmful substance” and its effects on other plants and soil microorganisms is also requested in Japan regardless of the characteristics of the inserted gene(s) (Table 3). Although these data are not obtained in the US, ecological interaction data are assessed qualitatively for every GM crop during the growing season. This study assesses plant interactions with insect pests and disease, as well as plant responses to abiotic stressors. The results of the ecological interaction study are relevant for assessing the release of harmful substances from GM crops and, if meaningful differences were detected between a GM crop and its conventional control further analysis may be needed to inform the ERA. Furthermore, more detailed and targeted NTO studies were conducted for lepidopteran insect-protected corn MON 89034 because insecticidal proteins such as Cry1A.105 and Cry2Ab2 expressed in MON 89034 could negatively affect the diversity and abundance of non-target arthropod communities including predators, parasitoids, and other ecologically important non-target arthropods. The assessment took into consideration several components, including the familiarity with the mode of action of Cry proteins, the activity spectra of the Cry1A.105 and Cry2Ab2 proteins, the expression levels of the two proteins in MON 89034, the environmental fate of the proteins, any potential interaction between the two proteins, and feeding tests of the two proteins or MON 89034 corn materials to representative NTOs. As the result of the comprehensive assessment of the potential impact of MON 89034 and the introduced proteins on NTOs and endangered species, it was concluded that environmental risk to these organisms from the use of MON 89034 was negligible (Monsanto Company 2006). These comprehensive studies to confirm the impact on NTOs and endangered species were not conducted for non-insect-protected events such as LY038 and MON 87460, because they do not have insecticidal activity.

As described above, there are some differences in the data requirements for GM corn and cotton between the US and Japan. However, additional data such as abiotic stress tolerance and release of harmful substance are obtained in the US depending on the characteristics of the inserted gene(s) and/or results obtained from the confined field trials.

Both the US and Japan evaluate plant characteristics that may be related to weediness potential regardless of the characteristics of inserted gene(s). For example, seed dormancy, plant lodging, and seed pod shattering are recognized as important characteristics related to weediness potential of soybean in the US (Horak et al. 2015). Seed dormancy would be required for a seed to over-winter or establish self-sustaining populations over several seasons. In addition, plant lodging and seed pod shattering could potentially be associated with aspects of seed dispersal. The mature seeds would need to be dispersed to favorable niches for the plant to function as a weed outside of cultivation or in an agronomic setting and not be harvested at the end of the growing season. In the US, these plant characteristics, including dropped ears, stalk lodged plants, yield and germination of harvested seed, are evaluated for each GM crop product regardless of the characteristics of the inserted gene(s) as a part of the agronomic/phenotypic evaluation. Similarly the characteristics related to seed productivity (e.g. number of grain rows), seed shattering, and germination of harvested seed are always evaluated in Japan (Table 3). As the seed shattering in Japan is compared between GM corn and non-GM control by visual analysis, no statistical comparison is conducted for this endpoint.

This paper evaluates the following selected plant characteristics for the assessment of weediness potential for LY038, MON 89034, and MON 87460 from the US: dropped ears (#/plot), yield (bu/a), stalk lodged plants (#/plot) and germination of harvested seed (%) (Table 4), and from Japan: number of grain rows, 100 grain weight (g), number of grains per ear and germination of harvested seed (%) (Table 5). All of these endpoints are recognized as important characteristics related to weediness potential of corn.

**Table 4:** Selected plant characterization for evaluating weediness potential in the US and Chile

	Test	Control^a^	Reference range	
			Min	Max
Dropped ears (#/plot)				
LY038—US (2002)	0.3	0.2	0.0	4.0
LY038—US (2003)	0.3	0.2	0.0	15.0
MON 89034—US (2004)	0.1	0.2	0.0	1.0
MON 89034—US (2005-1)	0.0	0.1	0.0	0.3
MON 89034—US (2005-2)	1.4	0.9	0.0	2.0
MON 87460—Chile (well-watered)^b,c^	0.0	0.0	0.0	0.0
MON 87460—Chile (water-limited)^b,c^	0.0	0.0	0.0	0.0
MON 87460—US (typical agronomic)^b^	0.1	0.1	0.0	0.7
Yield (bu/a)				
LY038—US (2002)	104.1	112.9	11.2	266.1
LY038—US (2003)	129.5	129.6	43.9	261.4
MON 89034—US (2004)	192.9	191.3	92.8	290.8
MON 89034—US (2005-1)	205.5	195.1	171.0	220.0
MON 89034—US (2005-2)	126.8	125.7	31.7	203.5
MON 87460—Chile (well-watered)	220.7	220.0	166.7	248.4
MON 87460—Chile (water-limited)	114.5*	86.7	56.4	167.6
MON 87460—US (typical agronomic)	170.2	165.3	143.6	213.4
Stalk lodged plants (#/plot)				
LY038—US (2002)	1.0	1.5	0.0	21.0
LY038—US (2003)	2.0	3.4	0.0	25.0
MON 89034—US (2004)	0.8*	2.4	0.0	6.0
MON 89034—US (2005-1)	0.1	0.3	0.0	2.3
MON 89034—US (2005-2)	9.6	5.4	0.0	49.0
MON 87460—Chile (well-watered)^c^	0.0	0.0	0.0	0.0
MON 87460—Chile (water-limited)^c^	0.0	0.0	0.0	0.0
MON 87460—US (typical agronomic)	5.5	5.1	0.3	7.7
Germination (%)				
LY038	98.5	99.0	94.0	100.0
MON 89034	94.2	95.3	78.0	100.0
MON 87460	98.7	98.4	93.3	98.0

Evaluation timing and description for these items are provided in Online Resource 1

* Indicates statistical difference between the test and the control (*p* < 0.05)

^a^For LY038, its negative segregant was used as a control

^b^Three different water management regimes used for the field trial of MON 87460 are: (1) well-watered treatments, (2) water-limited treatments, and (3) water managed according to typical local agronomic practices. The specifics for water management treatments are reported in Sammons et al. (2014)

^c^No statistical comparisons were made due to lack of variability in the data. The test was considered effectively not different from the control because the test and control mean values were identical

**Table 5:** Selected plant characterization for evaluating weediness potential in Japan

	Test	Control	Reference range^a^	
			Min	Max
Number of grain rows				
LY038-A	14.7*	15.9		
LY038-B	14.3*	16.9		
MON 89034	16.8	16.1	12.3	16.9
MON 87460 (well-watered)	14.00	13.70		
MON 87460 (water-limited)	13.26	12.68	–	–
100 grain weight (g)				
LY038-A	29.1	28.1		
LY038-B	30.7*	26.6		
MON 89034	29.3	30.3	22.3	43.9
MON 87460 (well-watered)	29.95	30.53		
MON 87460 (water-limited)	21.54	20.99	–	–
Number of grains per ear				
LY038-A	559.7	610.0		
LY038-B	584.1*	725.6		
MON 89034	663.6*	592.1	549.2	728.6
MON 87460 (well-watered)	614.67	559.96		
MON 87460 (water-limited)	249.85	159.88	–	–
Germination of harvested seeds (%)				
LY038-A	98.9	96.7		
LY038-B	97.8*	93.3		
MON 89034^b^	99.4	100.0	86.7	100.0
MON 87460 (well-watered)	99.50	98.00		

Evaluation timing and description for these items are provided in Online Resource 2

* Indicates statistical difference between the test and the control (*p* < 0.05)

^a^The reference range was determined from the minimum and maximum mean values of the non-GM controls used in previous confined field trials of the following GM corn varieties: DLL25 (1998), NK 603 (2000), MON 863 (2000), MON 810 (1996, 2001), MON 88001 (2002), MON 88012 (2002), MON 88017 (2002), LY038 (2004), MON 89034 (2006), MON 87460 (2010) and MON 87427 (2010)

^b^Statistical comparison was not conducted on germination data. However, statistical comparison was conducted on number of germinated plants and there was no significant difference between the test and the control (data not shown)

In the US, statistical differences were observed in MON 89034 (2004) and MON 87460 grown in Chile (Water-limited treatment) in the comparison of stalk lodged plants (#/plot) and yield (bu/a), respectively. The mean value of stalk lodged plants for MON 89034 (0.8) was, however, within the range of the value of the reference varieties (0.0–6.0) planted at the same locations. The mean value of yield for MON 87460 grown in Chile (114.5 bu/a) was also within the range of values of the reference varieties (56.4–167.6 bu/a) planted at the same locations (Table 4). The increase in yield for MON 87460 under stress conditions in Chile was expected and proved the efficacy of MON 87460.

In Japan, statistical differences between the GM crop and conventional control were observed in the comparison of number of grain rows, number of grains per ear, 100 grain weight (g) and germination of harvested seeds (%). However, when the mean values of GM events (number of grain rows: 14.7 and 14.3 for LY038-A and LY038-B, respectively, number of grains per ear; 584.1 and 663.6 for LY038-B and MON 89034, respectively, 100 grain weight: 30.7 for LY038-B, germination of harvested seeds: 97.8 for MON 89034) were compared with the range of the minimum and maximum mean values of the non-GM controls used in previous confined field trials (number of grain rows: 12.3–16.9, number of grains per ear; 549.2–728.6, respectively, 100 grain weight: 22.3–43.9, germination of harvested seeds: 86.7–100.0), all GM values were found to be within the reference ranges (Table 5).

**Fig. 1:**
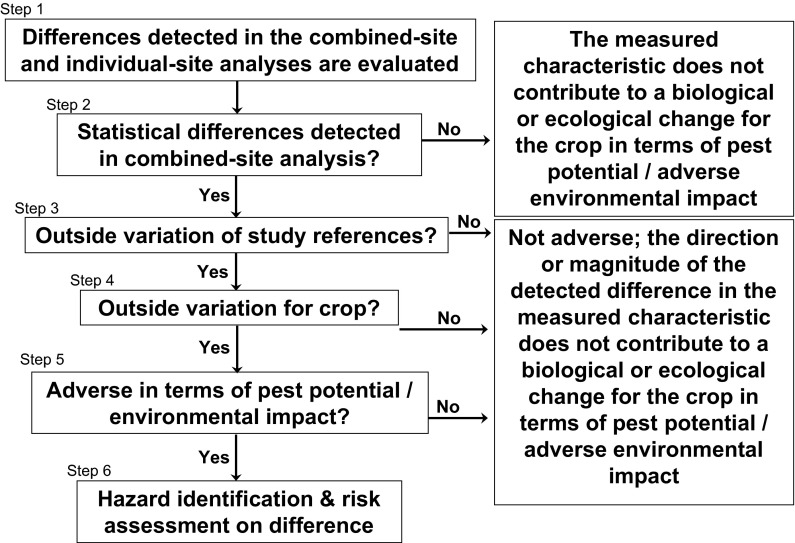
Decision diagram for interpretation of detected differences (adapted from Horak et al. 2015)

As described above, both the US and Japan use the concept of familiarity to interpret the statistical differences identified between the GM crop and non-GM control. However, it would appear that the US undergoes a more rigorous process than Japan to interpret statistical differences by conducting confined field trials at multiple locations and by obtaining the range of values of the reference varieties which were planted at the same locations. In the US, data from GM crops and non-GM controls are compared at a single location (pollen study) or across locations (germination study and growth and development studies) (Steps 1 and 2 in Fig. 1) as the initial steps (Horak et al. 2007, 2015). If a statistically significant difference between the GM crops and non-GM controls is detected, the mean value of GM crop is compared with the range of means obtained for the reference varieties grown in that study (Step 3 in Fig. 1). If the means of the GM crop is outside of the range of the means of the reference varieties, the GM crops’ mean characteristic value is considered in the context of published literature values for the characteristics for commercial varieties of the crop. If the GM crop mean value for a particular characteristic is outside the published characteristic value for commercial varieties, (Step 4 in Fig. 1), the characteristic would be assessed for the magnitude of the change and for whether or not it is adverse in terms of weediness potential or other ecological impact (Step 5 in Fig. 1) (Horak et al. 2007, 2015). In the case of confined field trials in Japan, there are often an insufficient number of non-GM control values to allow the development of a reference range. In this case, the GM crop mean value is directly assessed for the magnitude of the change and for whether or not it was adverse in terms of weediness potential or other ecological impact.

As described above, confined field trials in the US are conducted in diverse geographies representing a broad range of environmental conditions and agricultural ecosystems for which the crops is grown (Horak et al. 2015). Given the similarity of the assessment endpoints, such as the reduction in abundance of a valued species and the process by which this assessment is made, results from confined field trials in the US can be considered relevant to identify any potential ecological hazards of GM crops for FFP use in Japan. To facilitate data transportability more efficiently across different geographies, this paper advocates harmonization of protocols for confined field trials.

## Discussion

To consider the transportability of confined field trial data from cultivation countries to import countries for the ERA of GM crops from cultivation to import countries, it is important to have a clear understanding of the purpose of the confined field trial. While GM crops may exhibit different growth under different environmental conditions such as soil type and weather conditions, the purpose of confined field trials for ERA is not to describe GM crops in as much detail as possible in each of different environmental conditions. Rather, the purpose of confined field trials for GM crops is to identify whether any unintended and adverse changes occurred related to the ERA assessment endpoints (Raybould 2007). Additionally, for host crops which exhibit low weediness potential and have no sexually compatible wild relatives, a primal purpose of confined field trial is to identify any potential increases in weediness potential.

In addition, understanding the weediness potential of host crops is important when considering the transportability of confined field trial data from cultivation countries to import countries for the ERA. If the host crop has high weediness potential or a sexually compatible wild species exists in the import country, a necessity of confined field trials can be considered in the import country. The host crop in this situation could exhibit weediness characteristics in the receiving environment, if the effect of the trait is related to weediness characteristics. Recently, the acceptance of data generated in confined field trials has been advocated, if the agro-climatic zone where the confined field trials is conducted is demonstrably representative of the agro-climatic zone in those geographies to which the data will be transported (Garcia-Alonso et al. 2014). However, the strict similarity of environmental conditions does not seem to be necessary for testing highly domesticated crops such as corn and cotton to detect any changes related to weediness potential which is one of the primal purposes of confined field trial of GM crops as described above. Weedy characteristics are often complex and encoded by many genes, and most of the commodity crops, including those mentioned above, have lost many of the weed-related traits of their wild progenitors through domestication (OECD Environment Directorate 2013). In the cases of highly domesticated crops such as corn and cotton, these fundamental weediness characteristics are not considered to be readily altered under different environmental conditions such as different soil type and weather conditions. For example, corn is the most widely cultivated grain in the world, and it can be grown in areas roughly bounded by a northern latitude of 58° to a southern latitude of 40°, which includes most of the US, China, Brazil, Argentina, and European countries (Maruyama 1981; OECD 2003). To date there has been no report that corn has been able to establish and persist in unmanaged areas (e.g., roadsides) from seed or grain spilled during transportation. Furthermore, the confined field trial is usually conducted under managed conditions excluding abiotic and biotic stressors that might confound the difference between the GM crop and its control. Also soil fertility for the confined field trial can be optimized and uniformly managed for test, control and reference plants.

Moreover, evaluation of the case studies comparing the confined field trials of three GM corn events (LY038, MON 89034, and MON 87460) between the US and Japan shows that the US regulatory framework requires conducting confined field trials in more diverse geographies than Japan, representing a broad range of environmental and agronomic conditions. Given the similarity of the endpoints being assessed and the process by which this assessment is performed, confined field trials in the US can be considered relevant and robust for identifying potential ecological hazards for the ERA of GM crops in Japan.

As of February 2015, 73 GM plant events excluding stacked events have been granted environmental safety approval for either cultivation or import after conducting confined field trials in Japan (Japan Biosafety Clearing House (J-BCH) 2014b). Out of the 73 GM plant events, 59 of these (23 corn, 10 cotton, 12 soybean, eight canola, two alfalfa, two rose, one sugar beet, and one papaya event) also underwent confined field trials in the US and are currently de-regulated in the US (USDA Animal and Plant Health Inspection Service 2014). The results of confined field trials consistently reached a conclusion of no impact on biodiversity in both countries. These results support that confined field trials conducted under diverse geographic and environmental conditions in cultivation countries for highly domesticated crops are sensitive enough to detect any potential adverse changes which may be related to weediness potential.

Furthermore, GM crop developers generally produce hundreds or thousands of unique events to screen during early phases of the product development cycle. Throughout the screening process these events are evaluated and only those which meet specified criteria (e.g., acceptable molecular characterization, efficacy, and phenotypic and agronomic performance) are advanced towards commercialization (Prado et al. 2014) and undergo confined field trial testing for regulatory approvals. The extensive product development and evaluation process ensure that the likelihood for unintentional adverse effects from GM crop products related to weediness potential is very low.

In addition to the above points, it is important to consider the differences in exposure levels between cultivation and import countries when considering transportability of confined field trial data between these countries. Risk is a function of both hazard and exposure. Hazard is the inherent property of an object or process, or of an action that might lead to harm (e.g. toxicity), while exposure is a measure of interaction between the hazardous object or action and a specific entity (usually one that is protected or valued) (Roberts et al. 2014). When evaluating the likelihood and seriousness of harm to the environment following the cultivation of a GM crop, the ERA assumes 100 % exposure over an extended period of time. Exposure and potential impact are expected to be the highest under cultivation conditions. However, under use as FFP, the exposure is significantly lower because few, if any, GM crop plants are present in an environment (OECD Environment Directorate 2013; Roberts et al. 2014). Roberts et al. (2014) states that the low-exposures associated with import countries may not necessitate the kind of extensive characterization of potential hazard that normally accompanies risk assessment for large scale environmental introduction, such as release for commercial cultivation. So far there are only two countries, Japan and China, which require local confined field trials for GM crops intended for use as FFP (USDA Global Agricultural Information Network (GAIN) 2013). Although the EU imports a large amount of GM soybean and canola, mainly from Brazil and Canada, respectively, the EU clearly differentiates ERA of GM crops for the purposes of importation from those of cultivation due to differing exposure levels (EFSA Panel on Genetically Modified Organisms (GMO) 2010). For import applications, the EU accepts confined field trial data generated entirely in the countries in which these products are cultivated and grown (e.g. the US or Latin American countries). Similar to the EU, Korea imports a large amount of GM crop material, but does not request in-country field trials for import purposes (Rural Development Administration (RDA) 2014).

Based on the above considerations, we conclude that the data obtained from confined field trials of GM corn and cotton, regardless of the characteristics of the inserted gene(s), is transportable from cultivation countries to importing countries (e.g. from the US to Japan). In the case of host crops which have relatively high weediness potential and/or sexually compatible wild relatives in Japan such as canola and soybean, further considerations are required to decide transportability of confined field trial data. However, even for GM canola and soybean, the majority of the ERA data collected in the cultivation country confined field trial is still informative to the ERA conducted in Japan.

Finally, it is important that the ERA for GM crops is done as efficiently and effectively as possible to avoid needless duplication of studies, and to reduce unnecessary regulation in light of accumulated evidence and experience (Fedoroff et al. 2010; Raybould 2007). Application of transportability of confined field trial data of GM crops should be particularly beneficial to public sector product developers and small enterprises that develop GM crops but cannot afford to replicate redundant confined field trials (Garcia-Alonso et al. 2014). To facilitate more efficient transportability of confined field trial data across different geographies, this paper advocates harmonization of protocols. Efficient regulation advances biotechnology development while adequately assessing the risk associated with each product based on historical experience and scientific evidence.

## Electronic supplementary material

Supplementary material 1 (DOCX 13 kb)

Supplementary material 2 (DOCX 13 kb)
