# Supplementary material for: Transportability of confined field trial data from cultivation to import countries for environmental risk assessment of genetically modified crops
Source: Transgenic Res. 2015 Jul 3;24(6):929–44. doi: 10.1007/s11248-015-9892-6 (PMC4639567; doi:10.1007/s11248-015-9892-6)
Supplement: Supplementary file 1 — Supplementary material 1 (DOCX 13 kb) [file 11248_2015_9892_MOESM1_ESM.docx]

Evaluation Timing and Description of the Selected Plant Characterization Endpoints for Evaluating Weediness Potential in the U.S.

| **Characteristic Measured** | **Evaluation timing** | **Evaluation description (measurement endpoints)** |
| --- | --- | --- |
| Dropped ears | Pre-harvest | Number of mature ears per plot dropped from plants |
| Stalk lodged plants | Pre-harvest | Number of plants per plot broken below the ear |
| Yield (bu/ac) | Harvest | Harvested weight of shelled grain, adjusted to 15.5% moisture |
| Germination (at 20/30°C) | After 4 and 7 days | Seedlings that exhibited normal developmental characteristics and possessed both a root and a shoot. |

## Transportability of confined field trial data from cultivation to import countries for environmental risk assessment of genetically modified crops

Transgenic Research

Authors: Shuichi Nakai ・Kana Hoshikawa ・Ayako Shimono ・ Ryo Ohsawa

S. Nakai ・ K. Hoshikawa

Monsanto Japan Limited, Kyobashi Soseikan building 6F, 2-5-18, Kyobashi, Chuo-ku Tokyo, 104-0031, Japan

e-mail: [shuichi.nakai@monsanto.com](mailto:shuichi.nakai@monsanto.com)

Tel: +81-3-6264-4875

FAX:+81-3-3566-5411

A. Shimono

Faculty of Science, Toho University, 2-2-1 Miyata, Funabashi, Chiba, 274-8510, Japan

R. Ohsawa

Faculty of Life and Environmental Sciences, University of Tsukuba, 1-1-1 Tennodai, Tsukuba, Ibaraki, 305-8572, Japan
