# Supplementary material for: Transportability of confined field trial data from cultivation to import countries for environmental risk assessment of genetically modified crops
Source: Transgenic Res. 2015 Jul 3;24(6):929–44. doi: 10.1007/s11248-015-9892-6 (PMC4639567; doi:10.1007/s11248-015-9892-6)
Supplement: Supplementary file 2 — Supplementary material 2 (DOCX 13 kb) [file 11248_2015_9892_MOESM2_ESM.docx]

Evaluation Timing and Description of the Selected Plant Characterization Endpoints for Evaluating Weediness Potential in Japan

| **Evaluation Item** | **Evaluation timing** | **Evaluation description** |
| --- | --- | --- |
| Number of grain rows | After Dry | Number of grain rows at center of ear |
| Number of grains per ear | After Thresh | Number of grains in a primary ear |
| 100 grain weight (g) | After Thresh | Weight of 100 grains.from the center of ear (LY038) or by weighting 40 g of grains and then counting the number of grains to determine the average grain weight followed by multiplication of the grain weight by 100 (MON 89034 and MON 87460) |
| Germination rate of harvested seeds (%) | After 7 days (LY038), after 3 and 5 days (MON 87460), and daily from the next day of planting for 5 days (MON 87460) | Number of emerged plants/Number of planting harvested seeds |

## Transportability of confined field trial data from cultivation to import countries for environmental risk assessment of genetically modified crops

Transgenic Research

Authors: Shuichi Nakai ・Kana Hoshikawa ・Ayako Shimono ・ Ryo Ohsawa

S. Nakai ・ K. Hoshikawa

Monsanto Japan Limited, Kyobashi Soseikan building 6F, 2-5-18, Kyobashi, Chuo-ku Tokyo, 104-0031, Japan

e-mail: [shuichi.nakai@monsanto.com](mailto:shuichi.nakai@monsanto.com)

Tel: +81-3-6264-4875

FAX:+81-3-3566-5411

A. Shimono

Faculty of Science, Toho University, 2-2-1 Miyata, Funabashi, Chiba, 274-8510, Japan

R. Ohsawa

Faculty of Life and Environmental Sciences, University of Tsukuba, 1-1-1 Tennodai, Tsukuba, Ibaraki, 305-8572, Japan
